# The effectiveness of the puncture channel plugging for reduction of complications after CT-guided percutaneous transthoracic needle biopsy

**DOI:** 10.1038/s41598-023-38915-y

**Published:** 2023-07-29

**Authors:** Dong-xu Wang, Yu-guang Wang, Guo-xu Ding, Bo Li, Rui-nan Liu, Zhong-wei Ai, Yang Wang

**Affiliations:** 1 Medical Imaging Center, the Second Affiliated Hospital of Qiqihar Medical University, Qiqihar, 161006 Heilongjiang China; 2 Department of Pathology, Qiqihar Medical University, Qiqihar, 161006 Heilongjiang China

**Keywords:** Oncology, Cancer, Respiratory tract diseases, Respiratory signs and symptoms

## Abstract

The effect of plugging the puncture channel with a mixture of hemocoagulase injection on the complications of CT-guided percutaneous transthoracic need biopsy (PTNB) was discussed. The medical records of PTNB were retrospectively studied from June 2017 to May 2022. In the study, the puncture channel of 626 patients were blocked, while remain 681 patients’ were not. The Mantel Haenszel method performed layered analysis and evaluated the correlation of adjusted confounding factors. The Odds Ratio and its 95% confidence interval were calculated using the Woof method. The incidence of high-level pulmonary hemorrhage was significantly reduced in patients with lesions ≤ 2 cm and different needle lengths. Patients with different pleural-needle tip angle and perineedle emphysema were blocked, and the incidence of pneumothorax and thoracic implants was significantly reduced. Through puncture channel plugging, the incidence of pulmonary hemorrhage, pneumothorax and thoracic catheterization of PTNB under CT guidance was reduced.

## Introduction

Accurate pathological diagnosis is absolutely necessary for the diagnosis of lung cancer^1,2^. The Korean Society of Thoracic Radiology proposed that the most common complications of PTNB were pneumothorax and pulmonary hemorrhage. Other complications like massive hemoptysis and air embolism may also lead to death^3^. In the past 5 years, some scholars have proposed methods such as fine needle aspiration biopsy (FNAB), autologous blood or saline injection, which can reduce the occurrence of complications^4,5^.

Although the PTNB with CT guidance is usually safe, complications like pneumothorax and pulmonary hemorrhage are also reported in different literatures with different incidence rate. It has been reported in the literature^6–8^ that the incidence of pneumothorax is 5.3–37%, the probability of chest tube placement is 1.7–10.9% and the incidence of pulmonary hemorrhage is 5–31%. Pneumothorax and pulmonary hemorrhage are caused by lung tissue damage. Several studies have shown that a variety of materials (such as autologous blood, hydrogel, etc.) can be used to block the puncture channel to reduce the occurrence of pneumothorax, but whether it can reduce the risk of pulmonary hemorrhage was not reported^9–11^. No studies have reported the effectiveness of using a mixture of gelatin sponge and hemocoagulase injection to block biopsy passages. The gelatin sponge can be prepared from pig skin, can be absorbed in the human body, and can be used for hemostasis and embolism. Hemocoagulase injection can be injected intravenously, subcutaneously or intramuscularly, or locally administered, which can stimulate both internal and external coagulation pathways^12^. Therefore, the mixture of hemocoagulase injection was considered as a plugging material for the puncture channel, which has better air tightness and hemostasis. The purpose of this study was to evaluate the effectiveness of applying a mixture of gelatin sponge and hemocoagulase injection to prevent pulmonary hemorrhage and pneumothorax in patients undergoing PTNB and to determine whether this approach reduced the rate of pleural catheterization.

## Materials and methods

### Ethical policy

All procedures were performed in accordance with the relevant guidelines and regulations^13,14^. On January 5, 2020, the study was approved and registered by the Ethics Committee of the Second Affiliated Hospital of Qiqihar Medical University(No. 37, Zhonghua West Road, Jianhua District, Qiqihar City, Heilongjiang Province, China, protocol no. 2020193), and complied with the Declaration of Helsinki. Because it is a retrospective study, the Ethics Committee of the Second Affiliated Hospital of Qiqihar Medical University have exempted the patient's right of informed consent, but the patient's name needed to be hidden.

### Study design

We retrieved the electronic medical records from June 2017 to May 2022, and got a total of 1720 records with the PTNB of CT-guided treatment. For the patients with an indeterminate or suspicious lung mass or nodule, the tissue biopsy diagnosis is needed. But some of them were not suitable for transbronchial biopsy or facing a failure in transbronchial biopsy. In this situation, PTNB was performed. Exclusion criteria included patients with lesions close to the pleura and patients with FNAB. The institution instituted a policy on puncture channel plugging in February 2020, and patient grouping is time-limited. Before this date, no puncture channel plugging was used and were identified as the control group; after this date, all patients received puncture channel plugging and were identified as the study group.

All biopsy procedures were performed by 2 physicians with over 15 years of experience (The number of non-vascular interventional operations by doctors was about 1200 per year, and the puncture technique was very proficient). According to the PTNB guidelines^14^, before the biopsy, all patients have stopped anticoagulation and antiplatelet drugs, and their blood routine and coagulation function need to be reviewed. Also, they need to meet the requirement of platelet count > 50 × 10^9^/L and the international normalized ratio < 1.5. All of patients should sign the informed consent form of PTNB.

### Instruments and equipments

During the 64‑slice spiral CT (Aquillion; Canon Medical Systems Corporation) guidance, the 18G semi-automatic biopsy needles and matching 17G coaxial needles (TSK Surecut; TSK Laboratory) were used. The scanning layer thickness was 5 mm, tube voltage was 100 kV and tube current was adopted automatic low current.

### Biopsy procedure

**Figure 1:**
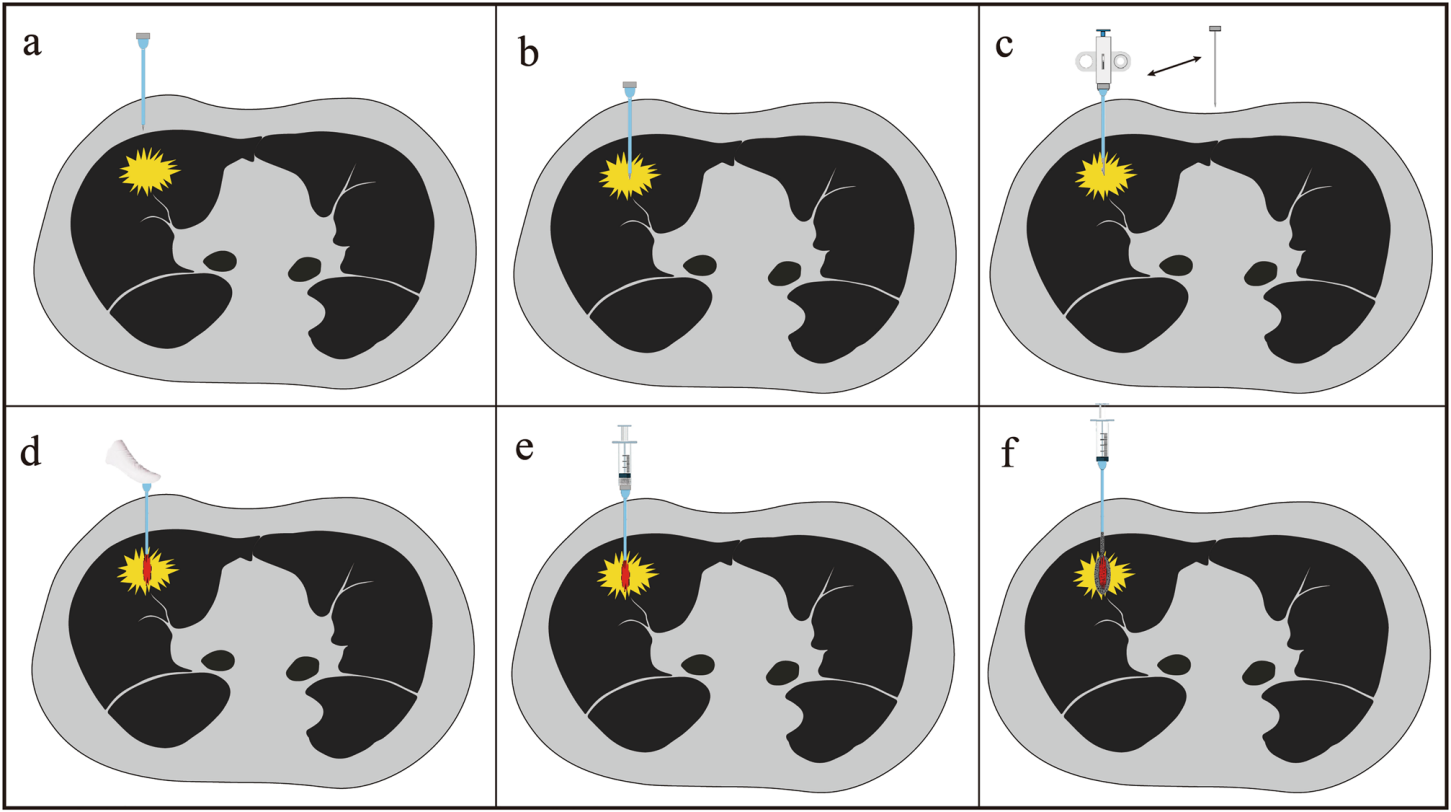
Schematic diagram of PTNB implementation steps: (**a**) After selecting the appropriate puncture point and needle path, CT scan is performed immediately as soon as the coaxial needle penetrates the extrapleura. (**b**) After adjusting the direction, the coaxial needle penetrates the edge of the lesion. (**c**) The needle core is taken out and it is put into the biopsy needle. (**d**) After obtaining the tissue, the tail of the coaxial needle is quickly plugged with the thumb. (**e**) The syringe containing the gelatin sponge slurry is connected. (**f**) Puncture channel plugging technique: immediately the coaxial needle is withdrawn the plugging material is injected into the pleura and lung parenchyma.

We performed the following procedures according to the guidelines of PTNB in China^14^. The patients were fasted with water for 6 h before the biopsy. The fence-like locator was fixed on the body surface, and the appropriate puncture point and needle direction were selected according to the depth and position of the lesion after scanning. According to the PTNB guidelines^14^, the angle between the pleura and needle tip should be close to 90° as much as possible, and a smaller distance to the lung tissue is favorable. All the operations were performed during the end of patient's expiration. The PTNB was performed after disinfection, draping, and 2% lidocaine local anesthesia. The puncture and plugging process is shown in Fig. 1 (produced by Adobe Photoshop 2018, https://ps.cdycst01.cn/?qhclickid=4c675f9ec9cbf5e4): After the coaxial needle reached the outside of the pleura, CT scan was performed immediately, and the coaxial needle direction was adjusted to penetrate into the lesion (to ensure a single pleural puncture); And then, the needle core was taken out and the biopsy needle was put into it. With which the first piece of tissue of 1 mm × 10 mm was cut. The biopsy tissue was immediately put into formalin fixative and it was sent to the pathology department. If the patients needed genetic testing, the biopsy needle was put into the coaxial needle again to cut a second piece of tissue of 1 mm × 10 mm. The puncture channel plugging technology is as following: the tail of the coaxial needle was blocked with the thumb as soon as the coaxial needle was withdrawn from the tissue, which would prevent air embolism caused by the air entering, and at the same time, the plugging material (5 ml of the gelatin sponge slurry) was injected to block the puncture channel to the pleura and lung parenchyma. The doctor controled the injection speed of the plugging material according to the length of the needle channel (the injection speed of the long needle channel was slow), so that all the gelatin sponge slurry was evenly deposited in the needle channel. After the puncture channel plugging, a chest CT scan of the patient was performed to ensure that there were no complications (Pulmonary hemorrhage and pneumothorax). After the puncture, the patients were observed in the ward for 24 h, and did not need to pierce the downward position. After monitoring the patient's vital signs for 3 h, a chest X-ray was taken. And the patient would be discharged next day if there were no complications. The asymptomatic patients with a pneumothorax less than 25% would not be discharged until an observation of 24 h. If the pneumothorax was between 25 and 40% of the asymptomatic patients, the thoracic tube would be not necessary, and the observation would be continued until the pneumothorax was stable or reduced. In the situation where the pneumothorax exceeded 40% for the asymptomatic patients, they should be detained for farther observation. Once the symptoms appeared, the respiratory department and thoracic surgeon jointly made a decision on whether to place a tube in the thoracic cavity. The size of the pneumothorax was calculated from the previous literature^15^.

**Figure 2:**
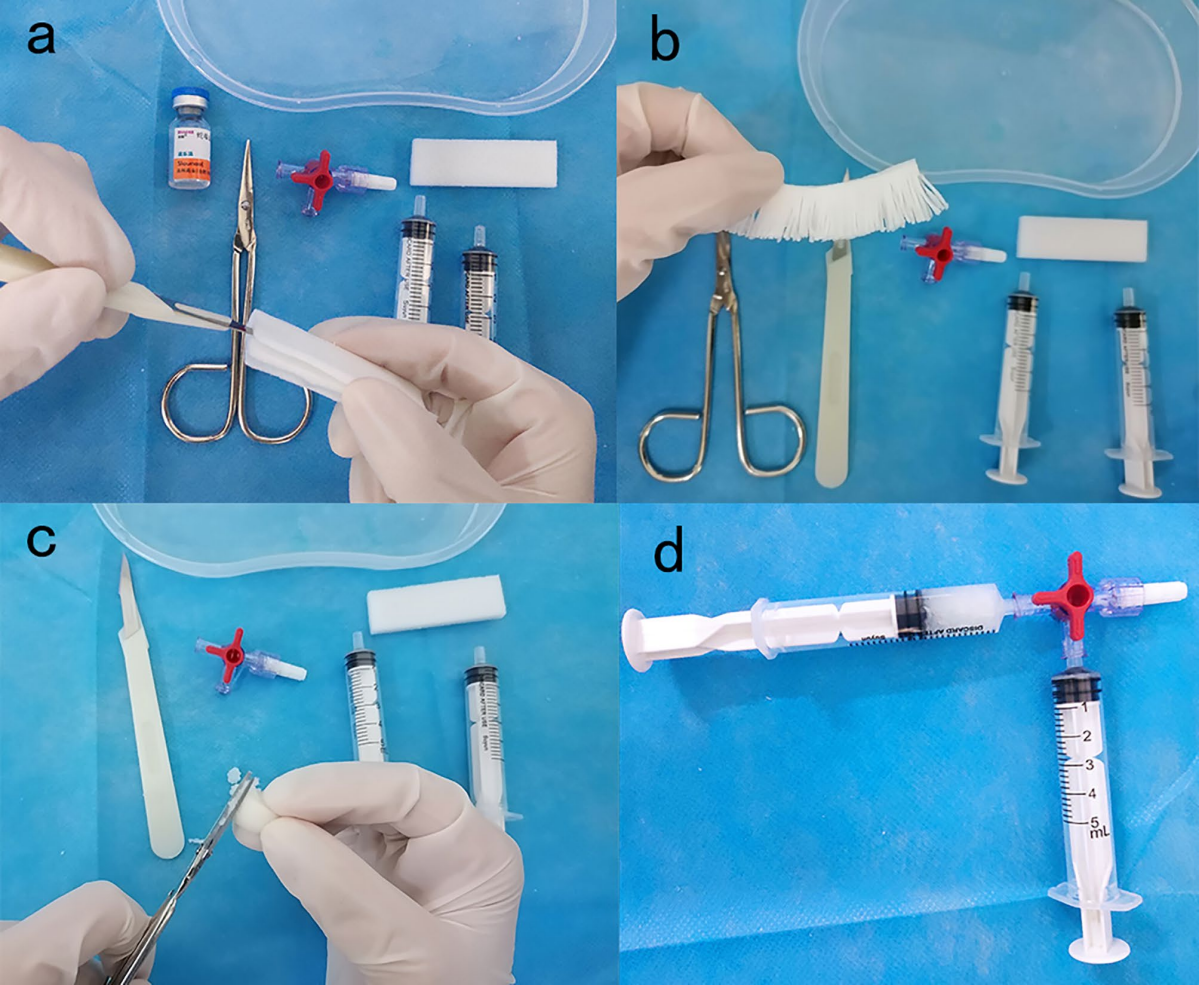
Preparation steps of gelatin sponge slurry: (**a**) The gelatin sponge is divided into three layers. (**b**) The gelatin sponge is cut into strips of 1–1.5 mm width. (**c**) The gelatin sponge is soaked in saline and rolled into a cylinder, and cut into a cube of 1–1.5 mm. (**d**) It is put into a 5 ml syringe and connected to a syringe with 1 mL of hemocoagulase injection through a three-way valve, and a flocculent gelatin sponge slurry is available after mixture.

Referring to the previous literatures^16–18^, steps of the preparation of the plugging material (Fig. 2): One piece of absorbable gelatin sponge (specification: 60 mm × 20 mm × 5 mm, Jiangxi Xiangen Medical Technology Development Co., Ltd.) were divided into three layers from the side with a blade and the thickness of each layer was about 1.6 mm. It was pressed by hand and cut into thin strips with a width of 1–1.5 mm. Moisten it with saline, it was rolled into a cylinder and cut into a cube of 1–1.5 mm. In the above process, one piece of the gelatin sponge (specification: 60 mm × 20 mm × 5 mm) should be cut into 1666 particles, and it would be put into the syringe. The syringe was connected to another syringe with 1 mL of hemocoagulase injection (specification: 1 ml:1 unit, Zhaoke Pharmaceutical Co., Ltd, instructions: General bleeding, 1–2 units of hemagmase injection for adults) and the 4 mL saline syringe through a three-way valve, through which a flocculent gelatin sponge slurry (5 ml) was acquired after mixture.

### Definitions and measured variables

Pulmonary hemorrhage is defined as newly-appearing ground glass density or consolidation after PTNB. It is a consensus that the severity of pulmonary hemorrhage should be categorized according to the grading scheme. Based on a previous hemorrhage grading scheme^19^, the categories include: Grade 0 is defined as no pulmonary hemorrhage; the pulmonary hemorrhage is limited to the needle path and within 2 cm around the lesion is considered to be Grade 1; Grade 2 is defined as that the pulmonary hemorrhage area exceeds 2 cm around the lesion and occurs only on one lobe; For Grade 3, the pulmonary hemorrhage is beyond the puncture lobe; Grade 4 is hemothorax. Grade 0–1 is a low-grade pulmonary hemorrhage and the grade 2–4 is high-grade pulmonary hemorrhage. The pleura-needle tip angle^19^ is defined as the acute angles between the puncture needle and the pleural tangent line, which is classified as 90° and less than 90°. We recorded the patient’s demographic characteristics, smoking history, lesion locations, lesion size, intraoperative position, whether there was emphysema near the needle track, the length through the lung tissue (measure the distance from the pleura to the lesion along the needle track), and the pleura-needle tip angle, pulmonary hemorrhage, hemoptysis, pneumothorax and chest tube placement. We obtained the histopathological results by reviewing the final pathology report of the specimens, and divided them into two categories: benign and malignant. If the lesion was surgically operated on, the pathological result was determined by the pathological report. If the biopsy was a malignant tumor, benign tumor or infection with a clear pathogen, it was a clear pathological result. If the nodule remains stable for more than 2 years, the lesion shrank after treatment (at least 3 months) or remained benign after a second biopsy, the lesion was considered benign. All data collection and image evaluation were carried out by a radiodiagnostic imaging doctor (not the surgeon who performed the operation) with more than 5 years of experience. Radiodiagnosis doctors are blind when evaluating CT images.

### Statistical analysis

Statistical analysis was performed using SPSS v18.0 software (SPSS, Inc.). The data was presented as the mean ± standard deviation for continuous variables. Data on categorical variables were presented as n-values and percentages. Data were compared using Student’s unpaired *t* tests or Mann–Whitney test for continuous variables and χ^2^ or Fisher’s exact test for categorical variables. According to previous literature^20^, plugging/non-plugging was used as explanatory variables, pulmonary hemorrhage, pneumothorax, and thoracic implantation as dependent variables were used for Pearson χ^2^ test, and the Mantel Haenszel method performed layered analysis and evaluated the correlation of adjusted confounding factors. The Odds Ratio and its 95% confidence interval were calculated using the Woof method. P < 0.05 had statistical differences.

### Ethics approval and consent to participate

All procedures performed were in accordance with the Declaration of Helsinki and the study was approved by the Ethics of the Second Affiliated Hospital of Qiqihar Medical University [approval no.2020193]. Because it is a retrospective study, the Ethics of the Second Affiliated Hospital of Qiqihar Medical University have exempted the patient's right of informed consent, but the patient's name needs to be hidde.

## Results

### Patients’ characteristics

**Figure 3:**
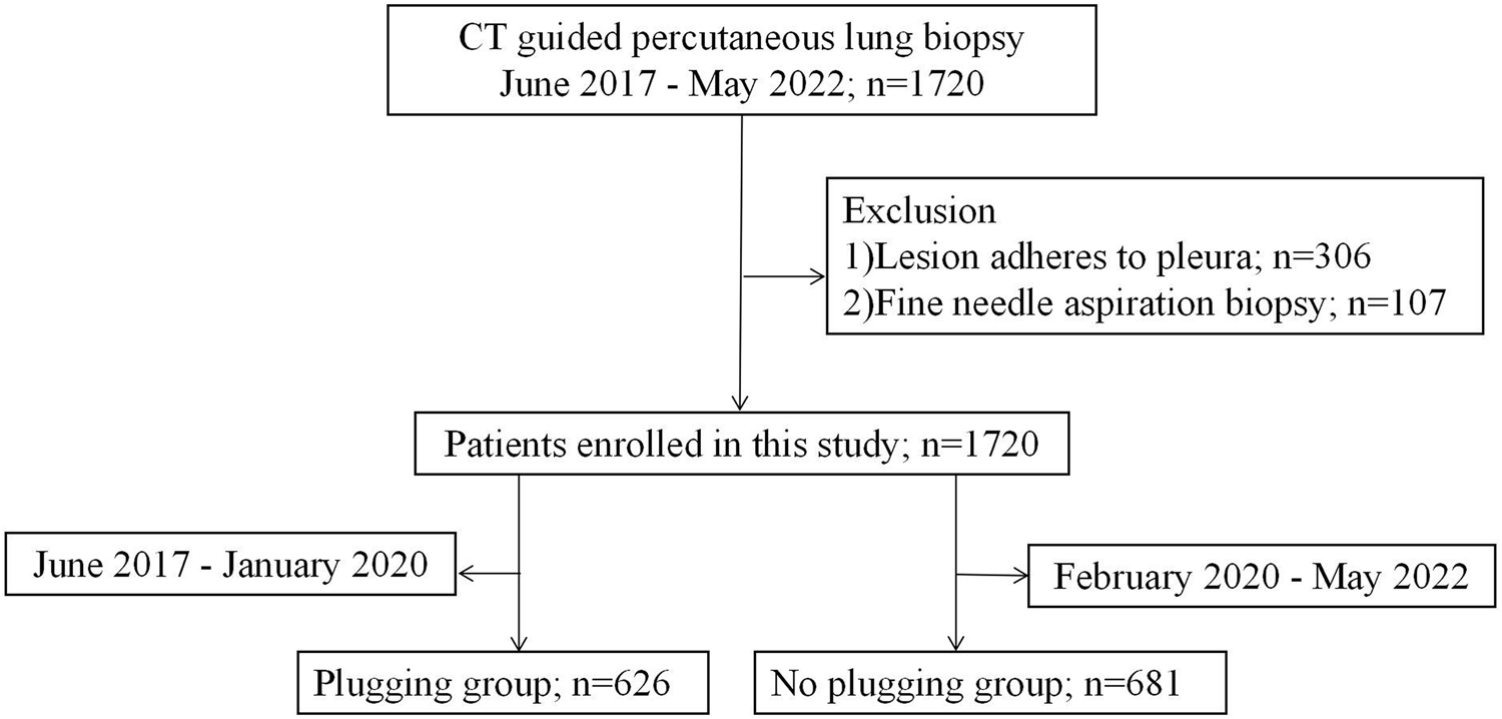
Flow chart of patients enrolled in the study.

306 patients with lesions close to the pleura (These patients were excluded who were without passing through the lung tissue so that it was impossible to plug the acupuncture tract.) and 107 patients with FNAB were excluded. The final study population included 1720 patients. The mean age ± standard deviation of patients was 63.13 years ± 10.49 (range, 20–94 years). A summary of the study population was shown in Fig. 3. There were 626 patients in the puncture channel plugging group. There were 681 patients in the non-puncture channel plugging group (Table 1).

**Table 1:** Demographical characteristics of the study population.

Patient groups	Plugging n = 626*	No plugging n = 681*
Demographics		
Age (years)^&^	63.28 ± 10.54	63.00 ± 10.46
Sex (male/female)	331 (52.88)/295 (47.12)	389 (57.12)/292 (42.88)
Smoke (yes/no)	57 (9.11)/569 (90.89)	69 (10.13)/612 (89.87)
Lesion location		
Right upper lobe	170 (27.16)	177 (25.99)
Right middle lobe	43 (6.87)	54 (7.93)
Right lower lobe	140 (22.36)	157 (23.05)
Left upper lobe	149 (23.80)	149 (21.88)
Left lower lobe	124 (19.81)	144 (21.15)
Lesion diameter (mm)^&^	33.67 ± 16.15	34.90 ± 17.82

*Except where indicated, data are numbers of procedures, with percentages in parentheses.

^&^Data are means ± standard deviations.

### Procedure characteristics and complication

**Figure 4:**
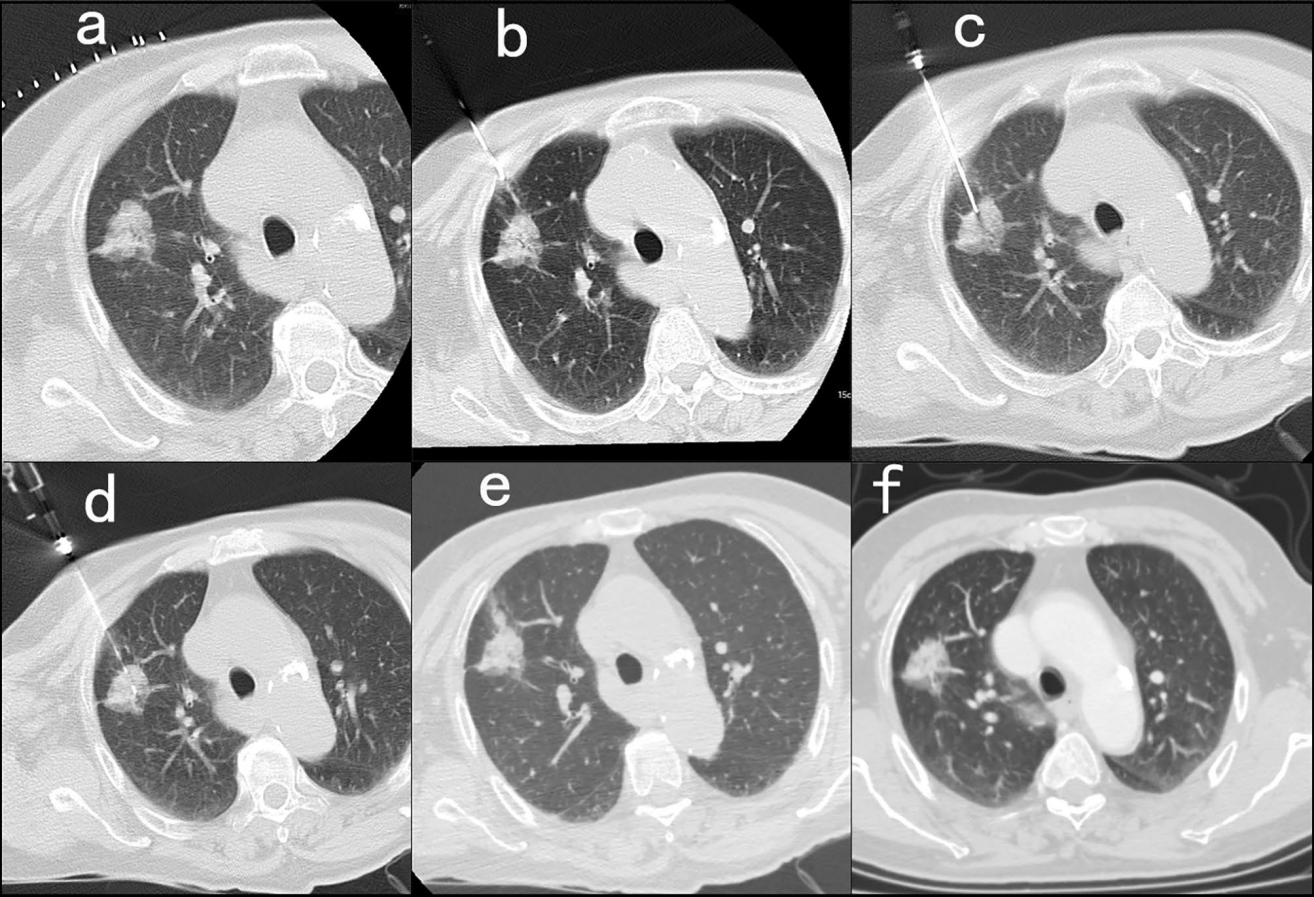
The process of plugging the puncture channel of a 64-year-old female patient: (**a**) The patient lies on the CT scan bed on her back, and a fence-like locator is placed on the skin surface. (**b**) The coaxial needle is punctured to the outside of the pleura. (**c**) After adjusting the direction, the coaxial needle reaches the edge of the nodule of the right upper lobe. (**d**) An 18G biopsy needle is inserted to cut the tissue. (**e**) When the coaxial needle is withdrawn, the plugging material is injected into the pleura and lung parenchyma, and the gelatin sponge slurry forms a striped shadow indicating high density. (**f**) The gelatin sponge slurry disappeared after 31 days, and the pathology report is lung adenocarcinoma.

In the plugging group, 19 cases (3.04%) underwent secondary biopsy, and the final diagnosis was 112 cases (17.89%) of benign lesions and 514 cases (82.11%) of malignant lesions. The puncture time (from the planned CT scan to the post-plugging CT scan) was 20.72 ± 2.85 min, and the radiation dose was 604.71 ± 62.22 mGycm. In the non-plugging group, 22 cases (3.23%) underwent secondary biopsy, and the final diagnosis was 125 cases (18.36%) of benign lesions and 556 cases (82.64%) of malignant lesions. The puncture time (from the planned CT scan to the post-plugging CT scan) was 20.46 ± 2.92 min, and the radiation dose was 598.93 ± 77.62 mGycm (Table 2). For the patients whose puncture channel was plugged, 109 of them had a CT scan during the continued 30 to 37 days after the procedure, and no images of gelatin sponge slurry were seen. Figure 4 showed a patient of lung adenocarcinoma with puncture channel plugging procedure. It was observed that the gelatin sponge slurry formed a blocked track in the lung tissue. After 31 days, CT showed that the gelatin sponge slurry was disappeared.

**Table 2:** Comparison of procedure characteristics and complication among the two patient groups.

Patient groups	Plugging n = 626*	No plugging n = 681*
Procedural characteristics		
Patient positioning		
Supine	344 (54.95)	347 (50.95)
Prone	57 (9.11)	75 (11.01)
Lateral recumbent	225 (35.94)	259 (38.03)
Needle tract length (mm)^&^	16.79 ± 12.16	15.81 ± 11.40
Pleural-needle tip angle (90°/ < 90°)	381 (60.86)/245 (39.14)	437 (64.17)/244 (35.83)
Number of pathological tissues (1/2)	374 (59.74)/252 (40.26)	432 (63.44)/249 (36.56)
The inflammation around the needle (yes/no)	43 (6.87)/583 (93.13)	50 (7.34)/631 (92.66)
Perineedle emphysema (yes/no)	56 (8.95)/570 (91.05)	49 (7.20)/632 (92.80)
The puncture time (min)	20.72 ± 2.85	20.46 ± 2.92
The radiation dose (mGycm)	604.71 ± 62.22	598.93 ± 77.62
The secondary biopsy (yes/no)	19 (3.04)/607 (96.96)	22 (3.23)/659 (96.77)
Pathology (benign/malignant)	112 (17.89)/514 (82.11)	125 (18.36)/556 (81.64)
Complication		
Hemorrhage (higher-grade/no or lower-grade)	82 (13.09)/544 (86.91)	127 (18.65)/554 (81.35)
Hemoptysis	11 (1.76)/615 (98.24)	13 (1.91)/668 (98.09)
Pneumothorax (yes/no)	95 (15.18)/531 (84.82)	140 (20.56)/541 (79.44)
Thoracic catheterization (yes/no)	16 (2.56)/610 (97.44)	33 (4.85)/648 (95.15)

*Except where indicated, data are numbers of procedures, with percentages in parentheses.

^&^Data are means ± standard deviations.

Analysis of the association between plugged puncture channels and pulmonary hemorrhage, pneumothorax and thoracic tubes According to the lesion size and needle tract length, Pearson Chi-square test showed that there was a statistical correlation between plugging and the occurrence of pulmonary hemorrhage. After Mantel–Haenszel test, the stratified combination effect of lesion size and needle tract length was statistically significant, as shown in Table 3.

**Table 3:** Analysis of the relationship between plugging and pulmonary hemorrhage.

Factors	Category	Procedure (plugging (yes/no))	N	Hemorrhage		Pearson Chi-square test				Mantel–Haenszel test			
				Higher-Grade	No or Lower-Grade	χ^2^_pea_	P_pea_	OR_pea_	OR_pea_. 95% CI	χ^2^_MH_	P_MH_	OR_MH_	OR_MH_. 95% CI
Lesion diameter						11.149	0.001	0.598	0.441, 0.810				
	≤ 2 cm	Yes	251	41	210	14.919	< 0.001	0.437	0.285, 0.669	11.827	0.001	0.579	0.426,0.788
		No	259	80	179								
	> 2 cm	Yes	375	37	338	0.994	0.319	0.796	0.509, 1.247				
		No	422	51	371								
Needle tract length						8.328	0.004	0.642	0.475, 0.869				
	≤ 3 cm	Yes	299	15	284	9.238	0.002	0.399	0.217, 0.734	11.164	0.001	0.582	0.426,0.795
		No	368	43	325								
	> 3 cm	Yes	327	66	261	4.314	0.038	0.678	0.470, 0.979				
		No	313	85	228								

*Pea* Pearson Chi-square test, *MH* Mantel–Haenszel test.

According to the layering of pleural-needle tip angle and perineedle emphysema, the Pearson Chi-square test showed that there was a statistical correlation between plugging and the occurrence of pneumothorax. After Mantel–Haenszel test, the stratified merger effect of pleural-needle tip angle and perineedle emphysema was statistically significant, as shown in Table 4.

**Table 4:** Analysis of the relationship between plugging and pneumothorax.

Factors	Category	Procedure (plugging (yes/no))	N	Pneumothorax		Pearson Chi-square test				Mantel–Haenszel test			
				Yes	No	χ^2^_pea_	P_pea_	OR_pea_	OR_pea_. 95% CI	χ^2^_MH_	P_MH_	OR_MH_	OR_MH_. 95% CI
Pleural-needle tip angle						13.577	< 0.001	0.581	0.435,0.777				
	< 90°	Yes	251	50	201	6.964	0.008	0.574	0.379, 0.869	15.382	< 0.001	0.550	0.410,0.739
		No	238	72	166								
	90°	Yes	375	37	338	9.063	0.003	0.529	0.347, 0.804				
		No	443	76	367								
Perineedle emphysema						6.407	0.011	0.691	0.519, 0.921				
	Yes	Yes	53	27	26	5.453	0.020	0.383	0.169, 0.865	7.887	0.005	0.639	0.471,0.868
		No	52	38	14								
	No	Yes	573	68	505	4.670	0.031	0.696	0.500, 0.968				
		No	629	102	527								

*Pea* Pearson Chi-square test, *MH* Mantel–Haenszel test.

According to the layering of pleural-needle tip angle and perineedle emphysema, the Pearson Chi-square test showed that there was a statistical relationship between plugging and the occurrence of thoracic tube. After Mantel–Haenszel test, the stratified merger effect of pleural-needle tip angle and perineedle emphysema was statistically significant, as shown in Table 5.

**Table 5:** Analysis of the relationship between plugging and thoracic tube.

Factors	Category	Procedure (plugging (yes/no))	N	Thoracic catheterization		Pearson Chi-square test				Mantel–Haenszel test			
				Yes	No	χ^2^_pea_	P_pea_	OR_pea_	OR_pea_. 95% CI	χ^2^_MH_	P_MH_	OR_MH_	OR_MH_. 95% CI
Pleural-needle tip angle						9.312	0.002	0.380	0.200, 0.723				
	< 90°	Yes	268	10	258	5.949	0.015	0.390	0.178, 0.851	10.960	0.001	0.328	0.170,0.633
		No	221	20	201								
	90°	Yes	358	3	355	6.186	0.013	0.235	0.068, 0.811				
		No	460	16	444								
Perineedle emphysema						6.094	0.014	0.467	0.252,0.866				
	Yes	Yes	59	9	50	5.434	0.020	0.338	0.133, 0.859	9.188	0.002	0.353	0.182,0.684
		No	46	16	30								
	No	Yes	567	6	561	4.831	0.028	0.367	0.145, 0.930				
		No	635	18	617								

*Pea* Pearson Chi-square test, *MH* Mantel–Haenszel test.

After the test of Homogeneity of Odds Ratio by Breslow-Day and Tarone’s methods, the results showed that the interlayer OR values of lesion size, needle tract length, pleural-needle tip angle, and perineedle emphysema were homogeneous (P > 0.05) (Table 6).

**Table 6:** Test of homogeneity of odds ratio.

Comparisons	Stratified factors	Breslow-Day		Tarone’s	
		χ^2^	P	χ^2^	P
Plugging vs hemorrhage	Lesion diameter	3.640	0.056	3.639	0.056
	Needle tract length	2.155	0.142	2.155	0.142
Plugging vs pneumothorax	Pleural-needle tip angle	0.073	0.787	0.073	0.787
	Perineedle emphysema	1.784	0.182	1.783	0.182
Plugging vs thoracic tube	Pleural-needle tip angle	0.470	0.493	0.468	0.494
	Perineedle emphysema	0.015	0.902	0.015	0.902

## Discussion

The CT-guided PTNB is a relatively safe technique. But the lung tissue is filled with much air, blood supply is also abundant and around the blood vessels are often accompanied with bronchi distribution, it is prone to lead to pulmonary hemorrhage and pneumothorax may easily occur during lung biopsy^6^, which makes it particularly important to reduce the incidence of complications. This study retrospectively analyzed the effect of puncture channel plugging on complications. Compared with the previous literatures, we have improved plugging materials, and our study is the largest single-institution assessment of plugging biopsy access. The gelatin sponge slurry was prepared before the planned CT scan, and the plugging process could be completed in 1–2 s without increasing the operation time and radiation dose.

Gelatin sponge is a degradable, absorbable, non-toxic, non-antigenic and non-water-soluble hemostatic material. It has been used for a long time in surgical procedures and vascular plugging^21^. Its porous structure can absorb a large amount of blood, and has the functions of swelling and compressing hemostasis and sealing the puncture channel. Hemocoagulase injection is extracted from snake venom. It can promote both the endogenous coagulation pathway and the exogenous coagulation pathway. As a coagulant, it can be exploited to treat various bleeding diseases and the usage of common dose is usually safe^22–25^. Slezak et al.^26^ proposed that recombinant topical thrombin with a gelatin sponge carrier was a more effective hemostatic agent in a pig liver hemorrhage model. Therefore, we chose the mixture of gelatin sponge andhemocoagulase injection as the plugging material in our study.

At present, literatures^27,28^ have reported several techniques to reduce the occurrence of pneumothorax, such as puncture after the patient exhales, procedure on the side of the disease, and changing the angle between the puncture route and the pleura. In 1974, McCartney et al.^29^ first proposed that the occlusion method can be used to reduce the complications of pneumothorax. After that, scholars have continued to explore various plugging materials and plugging procedures. For example, Turgut et al.^10^ proposed that the incidence of pneumothorax of 91 patients was 14.1% after the puncture channel was blocked by autologous blood, while the incidence of pneumothorax of 171 patients without puncture channel plugging was 26.3%. Baadh et al.^30^ used hemostatic gelatin powder to block the puncture channel, and the probability of thoracic tube placement was reduced from 8.1 to 4%, but the disadvantage was that it was expensive. In our study, the puncture channel plugging procedure reduced the incidence of pneumothorax from 20.56% (140/681) to 15.18% (95/626), and the incidence of thoracic catheterization from 4.85% (33/681) to 2.56% (16/626). This may be related to the fact that the expansion of the gelatin sponge blocked the puncture channel. Graffy et al.^31^ used autologous blood to block the puncture channel, which reduced the incidence of pneumothorax from 44 to 30%, and the probability of thoracic catheterization from 7.7 to 3.7%. The complication probability of lung puncture in their study was slightly higher than our results, which may be because that their plugging material not completely blocking the channel, or their study sample size was small may also be a factor. Renier et al.^32^ reported that the usage of the mixture of gelatin sponge and saline to block the pleura, reducing the incidence of pneumothorax from 25.8 to 10% by reducing the inflow of air into the pleural cavity. However, the previous literature did not report how to reduce the incidence of pulmonary hemorrhage. In this paper, a mixture of gelatin sponge and hemocoagulase injection was used to block the puncture channel, which reduced the incidence of high-grade pulmonary hemorrhage from 18.65% (127/681) to 13.09% (82/626). This is because the compression of the gelatin sponge can stop bleeding and the hemocoagulase injection would promote blood clotting. In our study, the plugging puncture channel did not significantly reduce the incidence of hemoptysis, which may be due to the fact that plugging the puncture channel was ineffective for rapid bleeding. Our research results showed that the secondary biopsy rate of puncture channel plugging was 3.04% (19/626), and that of non-puncture channel plugging was 3.23% (22/681). The plugging step was carried out after taking the pathological tissue, and will not reduce the success rate of diagnosis.

We found that blocking the puncture channel for patients with lesion ≤ 2 cm can reduce the occurrence of high-level pulmonary hemorrhage. On the level of lesion size > 2 cm, the interlayer OR value was homogeneous, and the lesion size factor did not affect the overall effect of the association between congestion and pulmonary hemorrhage, that was, the implementation of plugging technology effectively reduces the risk of high-level pulmonary hemorrhage. At the same time, regardless of the length of the needle, the plugging technology will significantly reduce high-level pulmonary hemorrhage. Pleural-needle tip angle and perineedle emphysema were both risk factors for pneumothorax and thoracic tubation. We analyzed the data in layers and found that whether thepleural-needle tip angle was 90° or not and with or without perineedle emphysema, the plugging technology could significantly reduce the incidence of pneumothorax and thoracic vascularization.

We injected the gelatin sponge slurry into the lung tissue through a coaxial needle and the CT image showed strips with high density, which was consistent with the shape of the puncture channel. The gelatin sponge slurry sealed the damaged lung tissue and effectively prevented pulmonary hemorrhage and pneumothorax caused by lung injury. Although air embolism is a serious but rare complication^33^, manual injection of the rubber sponge slurry will not cause high pressure. Also plugging the tail of the coaxial needle with thumb will prevent air embolism caused by air entering. In our research, we found that the high density strips in the CT image disappeared after 30 days, indicating that the plugging material could be absorbed by the body.

Our study has the following limitations. First of all, the study was retrospective, but after a departmental policy was established in February 2020, all patients were treated with this technique and patient selection bias was the most minimal. Although this study was operated by two doctors with more than 15 surgical experience, and the technical level was almost stable, there may still be small technical deviations. Even so, prospective studies may be more appropriate to evaluate the technique. Secondly, in our study, there were no such complications as air embolism and death, so we cannot evaluate this rare complication. Third, like most studies on the subject of the puncture channel plugging, we did not compare other materials with the mixture of gelatin sponge and hemocoagulase injection.

## Conclusion

In short, we found that the mixture of gelatin sponge and hemocoagulase injection was not only easy to obtain and of low cost but also it was effective in plugging the puncture channel through coaxial needle injection. And the puncture channel plugging procedure was proved to be effective in reducing the incidence of high-grade pulmonary hemorrhage, pneumothorax and thoracic catheterization after PTNB.

## Data Availability

Correspondence and requests for materials should be addressed to the corresponding author D.W.

## Abbreviations

FNAB: Fine needle aspiration biopsy
PTNB: Percutaneous transthoracic needle biopsy
